# Effects of prolonged exposure to feedback delay on the qualitative subjective experience of virtual reality

**DOI:** 10.1371/journal.pone.0205145

**Published:** 2018-10-24

**Authors:** Loes C. J. van Dam, Joey R. Stephens

**Affiliations:** Department of Psychology, Faculty of Science and Health, University of Essex, Colchester, Essex, United Kingdom; University G d’Annunzio, ITALY

## Abstract

When interacting with virtual environments, feedback delays between making a movement and seeing the visual consequences of that movement are detrimental for the subjective quality of the VR experience. Here we used standard measures of subjective experiences such as ownership, agency and presence to investigate whether prolonged exposure to the delay, and thus the possibility to adapt to it, leads to the recovery of the qualitative experience of VR. Participants performed a target-tracking task in a Virtual Reality environment. We measured the participants’ tracking performance in terms of spatial and temporal errors with respect to the target in both No-Delay and Delay conditions. Additionally, participants rated their sense of “ownership” of holding a virtual tool, agency and presence on each trial using sliding scales. These single trial ratings were compared to the results of the more traditional questionnaires for ownership and agency and presence for both No-Delay and Delay conditions. We found that the participants’ sliding scales ratings corresponded very well to the scores obtained from the traditional questionnaires. Moreover, not only did participants behaviourally adapt to the delay, their ratings of ownership and agency significantly improved with prolonged exposure to the delay. Together the results suggest a tight link between the ability to perform a behavioural task and the subjective ratings of ownership and agency in virtual reality.

## Introduction

Virtual Reality (VR) is an increasingly popular tool for a very wide range of applications. Besides its entertainment value it is, for instance, being investigated as a tool for the treatment of pain [1] and for rehabilitation purposes [2, 3]. It has also become a tool to study the perception of self and body ownership and how the perception of self and others can be modified by placing a participant “inside” an avatar of another body in the virtual world [4–9]. To optimize these procedures, previous research has focussed on how to optimize the overall subjective VR experience in terms of presence (the sense of “being there” in the environment) and the possible “embodiment” of an avatar or tool in terms of a sense of “ownership” over the avatar/tool and a sense of agency of its movements. For instance, for the sense of presence effects of visual refresh rate, visual feedback delay, stereoscopic viewing, visual pictorial realism, multisensory cues and body representation have previously been investigated [10]. In these studies often relatively short VR exposures were used in order to test the various display conditions. However, most practical applications of VR will involve longer periods of being in a virtual world and how the subjective experience as measured by standard ownership, agency and presence questionnaires may vary with exposure time has hitherto received relatively little attention. Therefore, the present study focussed on how the subjective ratings for ownership, agency and presence might change with prolonged exposure to the virtual world. More particularly, the present study investigated how exposure can serve to overcome parameters such as feedback delays between moving our own real limb and the corresponding visually observed movement of the controlled object in VR that otherwise may have a detrimental effect on the VR experience.

To measure the subjective experience of VR we used traditional measures for ownership, agency and presence in the form of subjective ratings and questionnaires. The questionnaire used to obtain subjective ratings for ownership and agency in the present study is an adapted version of the ones traditionally used in the study of hand ownership and agency through, for instance, the Rubber Hand Illusion [11, 12]. For this illusion a rubber hand is placed in front of an observer while their corresponding real hand is hidden from view and held passive and stationary. Next, both the rubber hand and the real hand are simultaneously stroked to give the illusion that the visual touch on the rubber hand corresponds to the felt touch on the real hand. Thus the illusion is created that the rubber hand is one’s own. Using such visuotactile stimulations as in the Rubber Hand Illusion the ownership illusion has been shown to occur for rubber hands, full body dolls and mannequins [13–15] and even empty space [16, 17]. A similar approach has been used in VR and a strong sense of ownership, as measured using questionnaires, is for instance reported when the movements of a virtual or robotic hand match those of the real hand [18, 19]. Here we applied the same questionnaire methods to a much simpler spherical shape rather than the image of a hand, but included 3-dimensional hand movements in terms of position and orientation into the design rather than asking participants to stay fully still. That is, in the virtual world the participants received continuous online feedback about their hand position and hand orientation in the form of a red cursor ball in the absence of direct vision of their own hand. To allow for the abstract shape of the cursor ball in the present study some of the questions for ownership were altered to reflect the idea of a hand-held object or tool rather than ownership of a body-part.

A key factor however for participants to report a subjective sense of ownership in paradigms such as the rubber hand illusion is the synchrony between the visual and either passive tactile stimulation or active movement signals. In the active case, when the feedback about the movements of the hand is asynchronous with the actual movements, a reduction in the subjective reports of the sense of ownership as well as a detriment in the sense of agency of the seen movements is observed [12, 20]. In other words the multisensory nature and the synchrony between the visual and kinaesthetic senses are essential to create the illusion of subjective ownership of a fake/virtual limb, avatar or tool [13, 20–22]. Indeed perceived synchrony provides a strong cue that the sensory signals are causally linked [23–25]. Therefore, great effort usually goes into maintaining the multisensory correspondence in order to promote the subjective senses of ownership and agency. However, even small feedback delays in the system (e.g. transmission delays if used over a network) can lead participants to report a reduced sense of ownership and agency [20, 26, 27].

Similarly, feedback delays resulting in multisensory asynchronies affect subjective ratings of presence in the virtual world [28]. Presence relates to our feeling of “being there” in the virtual world and is thought to include the feeling of spatial presence (the sense of physically being in the virtual space), involvement (relating to the extent to which we pay attention to the virtual world and for instance loose awareness of the real world) and realism (how real the virtual world looks and behaves) [29]. Besides the felt ownership and agency of a virtual avatar or tool, the sense of presence is another qualitative subjective measure for how immersed we are in the virtual world. Like ownership and agency, this inherently subjective experience has often been approached using questionnaires in which participants are asked to report their experience, and we used the same approach with one of the standard questionnaires here [29] to measure the immediate effect of feedback delay on the presence ratings.

Using such subjective measures previous research has focussed on the parameters that either enhance or attenuate the subjective ratings in VR such as the visuomotor feedback delays. However, most studies exclusively focussed on the immediate effects of these parameters. For instance, the detrimental effect of feedback delay on ownership and agency was measured using relatively short exposures of only 2 minutes [20]. Prolonged exposure to the feedback delay may however lead to delay adaptation, which causes a shift in the perception of what appears simultaneous in terms of making a movement and observing the visual consequences of that movement [30–33]. Even in the absence of genuine delay-adaptation, i.e. a shift in perceived simultaneity, participants generally learn to cope with the delay to some extent and their performance at behavioural tasks will improve over time [33, 34]. It is quite possible that with delay adaptation and/or a behavioural improvement in task performance the subjective ratings for the qualitative experience of VR improve as well.

Here we investigated the effects of prolonged exposure to a feedback delay applied to a virtual cursor tool used in the virtual environment and assessed both the behavioural improvement in a target-tracking task over time as well as the potential changes in the subjective reports of tool-ownership, agency and presence. We expected that as time progressed participants would adapt to the delay and at the same time report a stronger sense of agency of the controlled object in the virtual world and a better overall subjective experience. It is important to note that the nature of this research does not allow the usage of the questionnaires that are generally used to assess these subjective senses [12, 29] since that would imply removing the participants from the virtual world during the exposure which would interfere with the delay-adaptation process. Therefore we applied the method of sliding scales through which participants could indicate the level of ownership, agency and presence whilst remaining within the virtual world. For subjective ratings of presence similar sliding scales have previously been used in both VR and theatrical movie settings [35–37]. Also for the sense of ownership a sliding scale (sometimes referred to as a Visual Analog Scale, VAS) has been used before [38]. However, to our knowledge these and other studies did not test the correspondence with the more extensive and currently established questionnaires. Therefore, before measuring the effects of prolonged exposure to the feedback delays, we established the correspondence between using our simple sliding scales within the virtual world to the scores obtained from the existing questionnaires that participants filled in after being removed from the virtual world.

## Methods

The study was approved by the Ethics Committee of the Department of Psychology at the University of Essex prior to data collection (ethics code: LVD1703).

### Participants

Fifteen volunteers participated in the study (7 males, 6 females, 2 gender non-conforming; all self-reported right-handed). The age range was 22 to 44 (M = 30.8, SD = 8.0). The participants were naive with respect to the purpose of the experiment and written informed consent was obtained from each participant.

### Apparatus

Participants were seated on an office chair with enough surrounding space to be able to move their arms around without obstruction. An Oculus Rift head-mounted display was used in conjunction with the Oculus Touch controllers in order for the participants to interact with the virtual environment. The virtual environment was based on sample code that is included with the Oculus SDK (“OculusRoomTiny_Advanced/ORT (Controllers)”) which was modified to suit the purposes of the current experiment. The dimensions of the virtual room were 20 by 4 by 40 units in the virtual space (where 1 unit roughly corresponds to 1 m in physical space) and the room included 3 walls (at the left, right and back of the observer’s starting point), ceiling and a floor. Moreover the room included a virtual screen (2 by 1 m at 4.5 m distance in front of the observer in VR space) on which instructions to the participant could be displayed. Direct3D was used to render the 3D content of the virtual environment.

### Stimuli

Participants performed a target-tracking task in the virtual environment. The target stimulus was a green ball with a 5 cm radius floating roughly 1 m above the floor. The movement of the target was created by summing 5 sinusoids for each of the x, y and z directions separately. Each of the sinusoids had its own frequency and the amplitude for each was 5 cm (this means the maximum deviation of the target in any direction was 25 cm in the rare instances that the peaks in all sinusoids coincided). The frequencies for the sinusoids were 0.09, 0.165, 0.195, 0.375 and 0.495 Hz [33]. The starting phases for each of the sinusoids were drawn randomly from a uniform distribution between 0 and 2*π* on each trial. The random starting phases were drawn separately for the x, y and z directions.

**Fig 1:**
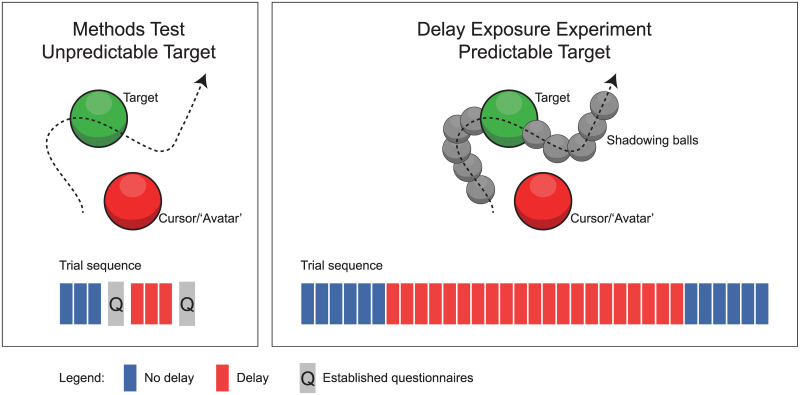
Schematic representation of stimulus and procedure of the experiment. The experiment consisted of two stages. In the first stage participants performed three trials for each of the No-Delay and Delay conditions after which they filled in the established questionnaires (left panel). In the second stage (right panel) participants first performed 6 pre-test trials without delay, then 21 adaptation trials with the added feedback delay of 200 ms and then 6 post-test trials without the delay. During the second stage of the experiment the target movement was rendered predictable by adding the shadowing balls which provided a 2 sec temporal window of the movement path of the target (up to both 1 sec ahead and 1 sec trailing the actual target position).

Delay-adaptation works best for a predictable target [33], however, the target by itself was relatively unpredictable in its movements. To make a predictable version of the target-tracking task [33], grey balls with a 2.5 cm radius shadowing the target were added to the display (Fig 1, right panel). There were 5 shadowing balls leading the target (to provide the predictable information necessary for delay adaptation) and 5 shadowing balls tailing the target. The shadowing balls moved such that they allowed a temporal movement path estimation window of 1 sec ahead of the target to 1 sec trailing the target.

Participants tracked the target using a red textured cursor ball which was controlled by the Oculus Touch controller for the right hand. In the virtual environment the red cursor ball was continuously being displayed at the location that corresponded to the location of the real right hand from the participants viewpoint. The cursor ball had a radius of 5 cm (the same as the target ball). This shape of the cursor was chosen to minimise the influence of roll, pitch and yaw on the position and movement estimates of the hand, though these rotations were rendered if these kind of movements were made and thus provided additional multisensory correspondence cues. The spherical shape of the cursor was chosen to ease performance measurements in terms of the centre to centre distance between the target and cursor balls. Note that using the red cursor ball, we exploited previous findings that even for such abstract shapes/tools a perhaps more fuzzy sense of ownership that includes tool-use can be reported, as long as participants are provided with sufficient experience of the sensorimotor correspondence [39].

Each target tracking trial lasted 20 seconds. After the 20 seconds were complete participants were presented with three questions using the screen within the virtual environment. The first question “how much did you feel like the ‘avatar’ was part of your body?” was used to measure the sense of ownership of the red cursor ball. The second question: “how much did it feel like you were in control of the ‘avatar’?” was used to measure agency for the cursor ball. Participants were informed that ‘avatar’ in these questions related to the red cursor ball. Finally, a third question “How present did you feel in the virtual environment?” was designed to measure presence within the virtual environment. The third question was explained to participants as a sense of “being there” in the virtual environment. These questions were chosen to match those commonly used on standard questionnaires for these subjective senses. For all three questions participants performed a scaling task and adjusted a marker on a continuous scale by using the thumbstick on the left Oculus Touch controller. The continuous scale ranged from “not at all” on the left side (roughly 1 m to the left of centre on the virtual scale) to “very much” on the right side (roughly 1m to the right of centre on the scale). A setting of “not at all” on the one side related to a value of -1. A setting of “very much” related to a value of +1. The virtual screen on which the questions were presented as well as the scale for the scaling task were presented at 4.5 m distance from the location of the target tracking task within the virtual space.

### Established questionnaires

In order to compare the results of the scaling task after each trial to the more established measures for ownership, agency and presence in the literature, we additionally used standard pen and paper questionnaires. As an established measure for presence we used the 14-item IPQ questionnaire [29]. The IPQ contains the sub-scales Presence (6 items: we combined the SP and PRES items in the IPQ to measure presence), Involvement (4 items) and Realism (4 items).

To measure ownership and agency we used a 16-item questionnaire [12]. This original questionnaire consists of 4 questions relating to ownership of the hand, 4 questions for agency as well as 4 control questions each for ownership and agency. For the present study references to the hand and hand movements in the original questionnaire were changed to the movement of the red cursor ball, since that was the visual stimulus used in the present experiment (see S1 Table). Therefore, “ownership” in the modified questionnaire does not necessarily reflect ownership of the red cursor ball as part of the body, but rather as a handheld object/tool. The only question amongst the 4 ownership questions that refers to body-ownership as such is the second question in S1 Table, which we kept for consistency with previous studies as well as with the scaling task performed within the virtual environment.

These questionnaires have become a standard for measuring such subjective feelings as presence in VR and ownership and agency. We therefore here applied them to the present case, despite the relatively abstract shape of the cursor ball. Both the IPQ and ownership and agency questionnaires consisted of 7 point Likert scales ranging from -3 to 3. To ease the comparison with the scaling task used for each trial within the virtual environment, the average scores obtained using the established questionnaires were divided by 3 to obtain a scale ranging from -1 to 1 as in the scaling task.

### Procedure

For each participant the experiment consisted of two stages (see Fig 1). The first part was a Methods Test Experiment to compare the results from our ownership, agency and presence scaling tasks on each trial with the corresponding more established questionnaires. Participants performed three trials without delay (No-Delay Condition) using an unpredictable target (i.e. without the shadowing balls). Participants started a trial by pressing the ‘A’ button on the right Oculus Touch controller after which they tracked the target ball for 20 sec using the right touch controller to control the red cursor ball. After 20 sec both the target and cursor disappeared and participants were prompted to provide ratings for their feelings of ownership, agency and presence, respectively, using the scaling task. The first question (ownership) along with the scale for the responses appeared automatically in the virtual environment once the 20 sec of the tracking task was complete. Participants adjusted the marker on the scale, using the thumbstick on the left Oculus Touch controller, until they were satisfied with their setting. To enter their rating they pressed ‘X’ on the left controller, at which point the next question was presented. This continued until all three questions were answered for that trial. At the start of each new rating the marker was positioned at the centre of the scale to prevent participants from simply rating all three questions similarly without adjusting the marker. Note that the thumbstick on the left Oculus Touch controller was used for the scaling task, which means that participants made these settings using the non-adapted hand and visuomotor adaptation usually does not to transfer between hands [40–42]. There was no added feedback delay whilst making these ratings. After both the tracking and scaling task were completed for all three trials, participants removed the oculus headset and filled in the established pen and paper questionnaires. They were instructed to relate their responses on these questionnaires to their experience of the three trials they had just performed.

Next the same procedure was repeated but now participants performed three trials with the added feedback delay of 200 ms (Delay Condition). While noticeable, this added delay of 200 ms has been shown to be small enough for delay adaptation [31, 33]. Again the tracking task was performed using an unpredictable target without shadowing balls to avoid adaptation [33]. Participants provided ownership, agency and presence ratings within the virtual environment at the end of each trial. Subsequently, after the three trials were completed, participants again removed the Oculus headset and filled in the established questionnaires, this time relating to the three 200 ms added feedback delay trials.

The second stage of the experimental session was the actual Delay Exposure Experiment in which we tested the effects of prolonged exposure to an additional 200ms feedback delay, and thus potentially delay adaptation, on the ratings for ownership, agency and presence in VR. The procedure on each individual trial was the same as for the Methods Test stage, except that now the predictable target including the shadowing balls was used in the target tracking task. The experiment followed a pre-test, adaptation, post-test paradigm and the sequence of trials was as follows (see also Fig 1, right panel). The experiment started with 6 no-delay trials (pre-test), after which 21 delay-adaptation trials followed. Finally to measure the potential aftereffects of delay-adaptation the experiment ended with a further 6 no-delay post-test trials.

### Analysis target-tracking task

On each trial the path of the target ball as well as the path of the cursor ball were recorded. These recordings were used to obtain the behavioural measures of adjusting to the added feedback delay of 200 ms. Before analysing participants’ tracking behaviour, the first 2 seconds for each trial were removed to avoid the period in which participants were still catching up to the target after the trial started from influencing the results. Next, we calculated the average distance between the target ball and the visual cursor ball within the virtual environment across the duration of each trial as our first behavioural measure. Additionally, we calculated the time-lag between the target and cursor paths in the tracking task, since this is the best measure for genuine delay compensation. The time-lag for each trial was obtained using a cross-correlation procedure on the target and cursor paths. The cross-correlation computes the correlation coefficients between the target and cursor paths whilst shifting one of them backwards and forwards in time relative to the other. The shift at which the correlation is maximal represents the time-lag between the two signals. The cross-correlation procedure was carried out for the x, y and z directions individually which means that for each dimension the correlation coefficient as a function of the time-shift was obtained. Next the resulting correlation coefficients as a function of the time-shift were averaged across the three dimensions before determining the time-shift for which the correlation between target and cursor paths was maximal. This shift represents the lag between the target and the cursor and thus will be referred to as our lag measure in the tracking task.

## Results

### Results methods test

**Fig 2:**
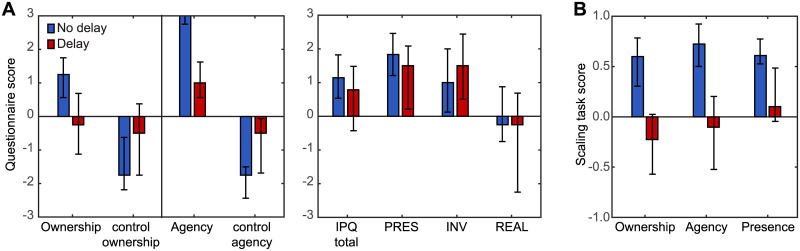
Questionnaire and scaling task results. (A) shows the results of the established ownership and agency as well as the IPQ questionnaires. The left panel shows the results from the ownership and agency questionnaire [12] the right panel shows the results from the IPQ [29]. IPQ results are shown as a total score and along the Presence (PRES), Involvement (INV) and Realism (REAL) subscales. (B) shows the median results for the scaling task. Blue bars represent the No-Delay condition, red bars the Delay condition. Bars represent medians and error bars represent the interquartile ranges across participants. The results confirm previous findings that an added feedback delay on the observer’s movement has detrimental effects on the subjective ratings for ownership, agency and presence.

Participants first performed two short blocks of trials, one each for the No-Delay and Delay conditions, and filled in the established ownership and agency questionnaire [12] (see S1 Table) and the IPQ [29] after completing the block of three trials for each condition. We calculated the scores for ownership, agency and presence by averaging the responses across the relevant questionnaire items. Additionally for the IPQ we calculated an overall average score across the Presence, Involvement and Realism dimensions. The results of the established questionnaires for the No-Delay and Delay conditions are presented in Fig 2A.

What can be seen is that for the subjective ratings of ownership and agency in particular the feedback delay has a strong detrimental effect on the subjective experience. That is, using Wilcoxon signed-rank exact tests significant differences were found between the No-Delay and Delay conditions in the questionnaire scores for ownership (W = 120; p = 0.000061) and agency (W = 120; p = 0.000061). The graph for the IPQ shows an apparent trend for a similar difference between the No-Delay and Delay conditions for the overall IPQ score. However, along the sub-scales Presence, Involvement and Realism, only Presence was significant but not at a level that would survive Bonferroni correction for multiple comparisons at *α* = 0.0125 (IPQ: W = 86; p = 0.147217; Presence: W = 95.5; p = 0.042; Involvement: W = 32; p = 0.36; Realism: W = 89.5; p = 0.096).

Besides filling in the established questionnaires after each block was complete, participants rated each individual trial whilst in the virtual environment in terms of ownership and agency of the red cursor ball as well as their sense of presence in the virtual environment, using the sliding scales. The sliding scales were a very quick method to obtain subjective ratings and we investigated whether they led to similar scores as the established questionnaires. For the scaling task within VR we averaged the ratings provided for each measure (ownership, agency and presence) across the three trials for each of the No-Delay and Delay conditions. The scores across participants for the scaling task are shown in Fig 2B. Using Wilcoxon signed-rank exact tests we found significant differences between the No-Delay and Delay conditions for all three subjective measures of ownership (W = 120; p = 0.000061), agency (W = 120; p = 0.000061) and presence (W = 110; p = 0.0026).

**Fig 3:**
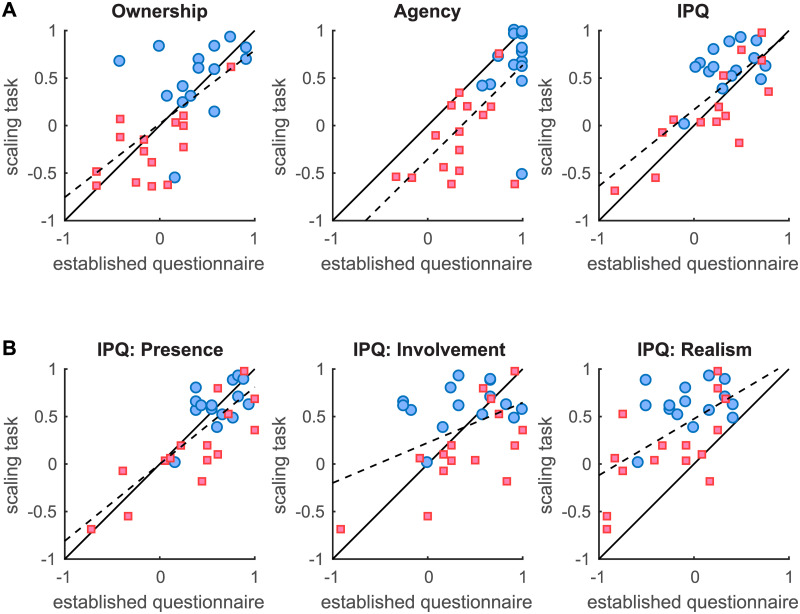
Relationship between the questionnaire and scaling task response modes. (A) shows the scatter plots linking the scores of the single trial sliding scales to the scores of the established ownership and agency as well as the IPQ questionnaires. The scatter plots in (B) shows the correspondence between each of the IPQ sub-scales and the presence ratings using the scaling task within VR. The average scores obtained using the established questionnaires were divided by 3 to obtain a scale ranging from -1 to +1 as in the scaling task for each trial. Each point in the plot represents an individual participant and for each participant there is a point both for the No Delay (blue disks) and the Delay (red squares) conditions. The continuous black line represents the prediction of both types of scores (sliding scales and established questionnaires) being equal. The dashed black line represents the best linear fit using linear regression.

Taken together the established questionnaires and the scaling task provide very similar results, even though the scaling task seems to provide stronger evidence for an effect of the delay on the subjective ratings of presence. In principle both questionnaires and sliding scales also measure the exact same quantities, just using a different response mode (Likert scale average versus continuous scale). Therefore, provided that the results for both response modes are scaled such that their respective extreme values match, we expect a linear relationship between the response modes with a slope of 1 (after rescaling). To test this linear correspondence between the established questionnaires and the scaling task we therefore divided the questionnaire results by 3 to match the two scales in terms of their extremes. Next we determined the correlation between the scores from the scaling task and those from the establishes questionnaires as well as determined the slope of the linear relationship using linear regression. Fig 3A shows the scatter plots linking each rescaled score for the established questionnaires to the corresponding sliding scale rating. What can be seen is that there is a very good correspondence between the ratings participants gave using the sliding scales and their scores from the established questionnaires. The correlation between the scores for each measure is quite high (ownership: r(28) = 0.65; p = 0.000087; agency: r(28) = 0.69; p = 0.000022; overall IPQ: r(28) = 0.72; p = 0.000007). Moreover, the slopes of the linear regression lines are not distinguishable from 1 (ownership: CI_95_ = [0.43, 1.12]; agency: CI_95_ = [0.59, 1.39]; overall IPQ: CI_95_ = [0.50, 1.10]), indicating that both types of responses indeed measure the same quantities. The only notable difference between the two types of scores is that for agency participants seem to consistently provide slightly lower ratings using the sliding scale task compared to the established questionnaire (CI_95,offset_: [-0.64, -0.063]) without breaking the unity correspondence.

We furthermore analysed the correspondence between the presence sliding scale ratings and each of the IPQ sub-scales. It appears that the sliding scales as used in the present task were relatively selective for the IPQ Presence sub-scale (Fig 3B). That is, even though all IPQ sub-scales correlate with the presence sliding scale ratings (IPQ Presence: r(28) = 0.80; p ≪ 0.001; IPQ Involvement: r(28) = 0.46; p = 0.011; IPQ Realism: r(28) = 0.59; p = 0.0006) only the regression slope for the IPQ Presence sub-scale is indistinguishable from 1 (IPQ Presence: CI_95_ = [0.57, 1.05]; IPQ Involvement: CI_95_ = [0.10, 0.74]; IPQ Realism: CI_95_ = [0.28, 0.91]).

In short, these results show that the sliding scales lead to very similar results as the more established questionnaires. The great advantage of the sliding scales is that they represent a very quick assessment method for which ratings can be provided without having to be removed from the virtual world. This makes them more suited to measure changes in the subjective ratings of ownership, agency and presence in prolonged exposure approaches.

### Results delay exposure experiment

**Fig 4:**
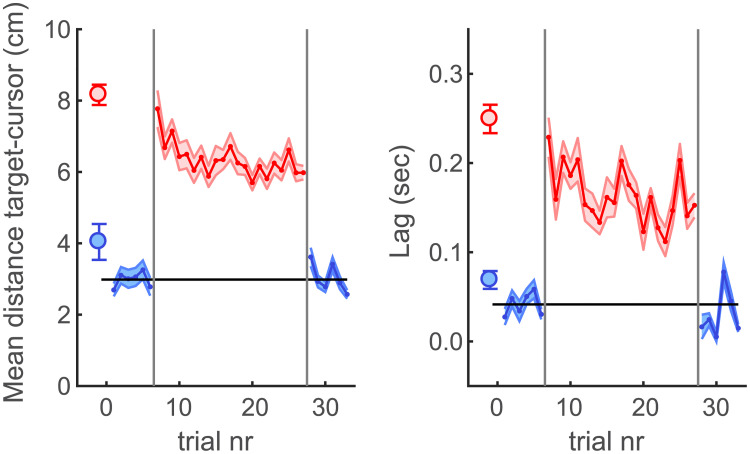
Behavioural results of the tracking task. The left panel shows the average distance between the target and cursor across the last 18 seconds for each trial. The right panel shows the visual movement time lag between the target and cursor. Blue lines (mean across participants) and shaded areas (standard error of the mean) represent the results for the no-delay trials during the pre- and post-test in the experiment. Red lines and shaded areas represent trials during the exposure phase in which the additional feedback delay of 200ms was present. The black horizontal line represent the average across the first six trials in the experiment (no-delay pretest) as a visual reference for investigating both direct effects of the added feedback delay as well as potential adaptation aftereffects in the post-test. The vertical grey lines represent the beginning and end of the delay exposure phase. The blue and red circles on the left-hand side represent the average results for the No-Delay and Delay conditions in the Methods Test part of the experiment for comparison.

In the Delay Exposure Experiment of this study we investigated the effect of prolonged exposure to an added feedback delay on both behavioural performance as well as the ownership, agency and presence ratings from the trial-by-trial scaling task. The behavioural target tracking performance was investigated in terms of the spatial differences between the target and the cursor as well as the time-lag between the target and cursor movements. The results of these behavioural measures are shown in Fig 4. The results show that the added feedback delay has an immediate detrimental effect on tracking performance. That is, when the delay is introduced (first delay trial) both the mean spatial distance as well as the lag between the target and cursor significantly increase compared to the average of the 6 pre-test trials (distance: t(14) = 10.1; p ≪ 0.001; lag: t(14) = 9.9; p ≪ 0.001). With prolonged exposure to the delay the results for both measures decrease again (i.e. tracking is improved). For both the spatial distance as well as the temporal lag this improvement is highly significant (distance: t(14) = 4.70; p = 0.0004; lag: t(14) = 4.13; p = 0.001) when comparing the first delay trial with the last delay trial. Note though, that of the two measures the lag appears to be more variable over time and upon debriefing some participants indicated that they used the strategy of tracking one of the shadowing balls instead of the target ball. This mix of automated adaptation and strategy to deal with the delay appears to have led to relatively noisy results in the temporal lag measure in particular.

In short, during delay exposure the behavioural performance in terms of both spatial distance and temporal lag recovered to some extent. However, for neither measure the recovery was complete. That is, for the spatial distance as well as the temporal lag the performance appears to asymptote at a level that is significantly different from the pre-test no-delay trials (comparing the mean across the last 6 delay trials with the 6 no-delay pre-test trials: distance: t(14) = 17.8; p ≪ 0.001; lag: t(14) = 11.2; p ≪ 0.001). This shows that exposure to the delay leads to only a partial recovery of the behavioural performance.

To test for potential behavioural aftereffects of delay adaptation we compared the results of the first post-test trial with the average across the 6 pre-test trials. We only included the first post-test trial, since deadaptation to the condition without the delay is often quite fast [33], as can also be observed in the present results, and we did not include top-up adaptation trials during the post-test. We found that in terms of the distance to the target behaviour appears to be worse again upon removal of the delay (distance: t(14) = 2.77; p = 0.015), whereas the aftereffect for the more noisy lag measure failed to reach significance (lag: t(14) = 1.99; p = 0.066).

To summarise the behavioural results, the added feedback delay led to a large immediate detrimental effect on task performance. However, with prolonged exposure to the delay, tracking performance increased in both the spatial and temporal sense, eventually even leading to a small aftereffect for the spatial distance measure when the delay is removed again.

**Fig 5:**
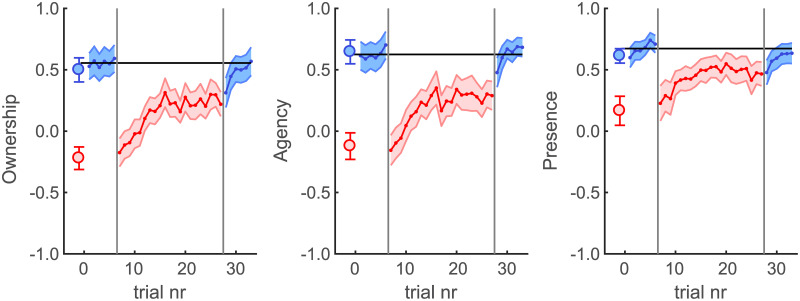
Scaling task results during the delay exposure experiment. From left to right the scaling task results are shown for ownership, agency and presence, respectively. Colour schemes are the same as for Fig 4.

We performed the same analyses using Wilcoxon signed-rank exact tests for the subjective ratings that participants provided on each trial for ownership, agency and presence (see Fig 5). For all three subjective measures the delay had a detrimental effect (ownership: W = 119; p = 0.00012; agency: W = 118; p = 0.00018; presence: W = 117; p = 0.00031) replicating the findings of our Methods Test when basing the analysis on the same scaling task scores (see Fig 2B). Note that this means that even for the presence measure we did find a significant effect of introducing the delay when using the scaling task both during the Methods Test Experiment as well as for the main Delay Exposure Experiment, whereas the significance of the presence measure as obtained using the IPQ questionnaire is somewhat questionable in the Methods Test Experiment.

During the course of the delay exposure the subjective ratings of ownership and agency significantly improved (ownership: W = 7; p = 0.0012; agency: W = 0; p = 0.000061). However, even though a trend is visible in the results for the subjective presence measure (Fig 5 right graph) the difference between the first and last delay trial was not significant in this case (presence: W = 34; p = 0.27). Likely this is because also the immediate effect of the delay is much smaller for presence than for ownership and agency since the link between the delay and the general appearance of the virtual world that participant are providing presence ratings for is less direct.

For all three subjective measures the recovery was not complete. Comparing the means for the last 6 delay trials and the 6 no-delay trials in the pretest shows that the recovery asymptotes at a level that is still significantly different from the corresponding pre-test values (ownership: W = 110; p = 0.0026; agency: W = 111; p = 0.0020; presence: W = 105; p = 0.0084). This is consistent with the results for the behavioural measures (spatial distance and temporal lag between target and cursor) for which the recovery was also incomplete.

Aftereffects for the subjective measures were very small and considering the multiple comparisons did not reach significance (ownership: W = 97; p = 0.035; agency: W = 77; p = 0.36; presence: W = 97; p = 0.035) even though trends of deadaptation are visible in all cases. This shows that the removal of the delay did not lead to strong renewed detrimental effects on these subjective measures.

Overall the results show improvement with prolonged exposure to the feedback delay, both in terms of behavioural performance as well as the subjective ratings for ownership, agency and to a lesser extent presence.

## Discussion

The purpose of the present study was twofold. First we investigated the possibility for a different method for obtaining subjective ratings for ownership, agency and presence in VR. The reasoning behind this was to find ways to assess these subjective experiences in a quicker way on any given trial compared to filling in multi-item questionnaires. A quick assessment of the subjective experiences of VR using ownership, agency and presence ratings would be more suitable for investigating effects over time without the necessity of removing participants from the virtual world. Previous studies have used similar scaling tasks as the one we presented here, but to our knowledge we are the first to compare them to the more established questionnaires that are commonly used to obtain subjective ratings for ownership, agency and presence in VR. We found that the results of the quick single trial scaling task used in the present study corresponded very well to the established questionnaires for ownership and agency [12] as well as presence [29]. Therefore the scaling task used provides a good alternative for situations in which a quick assessment is preferred or when it is essential that participants stay within the virtual environment when being tested on these measures.

Moreover, the second and main aim of the current study was to use the quick assessment method to investigate the effects of prolonged exposure to visual feedback delays in a target tracking task on the subjective experience in VR using the qualitative subjective ratings. We found that feedback delays had an immediate detrimental effect on all three subjective measures, confirming previous results [20, 26–28]. More importantly, however, we found that with prolonged exposure to the delay, and the corresponding improvement in tracking performance, the subjective ratings partially recovered. Both the detrimental effects and the recovery were strongest for the ratings for ownership and agency. These two subjective measures were also more directly affected by the delay since cross-modal correspondence and synchrony are very relevant cues for ownership and agency [11, 12, 20].

The effects of the feedback delay on the presence ratings were much smaller. One reason could be that the feedback delay is only one of multiple factors that will affect how present we feel in a virtual environment. For instance, the realism of our virtual environment was rated to be very low (Fig 2) and a lack of, for instance, pictorial realism can negatively affect the subjective ratings of presence [28, 43]. We also chose to only add the delay to the visual feedback of the hand and not to the updates of the visual display corresponding to the head movements that participants made. It is possible that delays on the movements of the head lead to a larger decrease in the subjective rating for presence as this will lead to a more global discrepancy between the bodily movements and the corresponding visual feedback. Moreover, a feedback delay on head movements will lead to the virtual world being perceived as less stable [28, 44]. However, visual delays of head movements in VR can lead to large conflicts between the vestibular and visual senses which is one of the major causes for motion sickness in general and this includes VR sickness [45, 46]. In our experiment participants were exposed to the virtual world for a relatively long period of time and motion sickness was something we wished to avoid. Therefore, in the present study we chose to apply the visual feedback delay only locally to the hand and not to all body movements. Future research could however make the comparison between the effects of local (e.g. hand only) versus globally applied (all movements or head movements) feedback delays and measure the effects on the subjective experience in VR. This would provide insights to the extent to which presence is a measure of the global VR experience.

Interestingly, presence was also the only measure for which there was a slight discrepancy in the interpretation between the quick sliding scale task within the virtual environment and the established IPQ questionnaire that was filled-in after exposure to the virtual environment. According to the established questionnaire the effect of the delay was very weak and does not survive Bonferroni correction for significance testing, whereas the sliding scale task indicated there was a significant detrimental effect of the delay on the subjective ratings of presence. This could of course be due to random variations in the participants’ responses since overall there was a very strong correspondence between both types of scores for measuring presence. However, a point to consider is that the two types of responses were made at different time-points. The quick sliding scale task was performed within the virtual environment that participants were rating. Instead, to fill in the IPQ questionnaire participants had to remove the VR headset, thus also removing them from the environment they were supposed to be judging. Potentially, this may have affected their ratings, which would also be an argument for having participants perform their judgements of the VR experience within the environment they are meant to judge, rather than rating the experience afterwards. Alternatively, however, it is also possible that the presence ratings made within the virtual environment where slightly biased toward the ratings for ownership and agency compared to the IPQ questionnaire, since rated using a very similar sliding scale. To further test the correspondence of these measures, it will be important for future research to use both types of approaches where possible. If within VR and post-VR judgements consistently differ across experiments this could potentially point to a relevant role of being in the virtual world while making presence judgements, but would also raise questions concerning the reliability of these subjective ratings.

However, a general word of caution is needed when interpreting the questionnaire and scaling task results. In the present study we used the questionnaires that are commonly used in the study of ownership, agency and presence in a somewhat adapted or completely unmodified form. These questionnaires have become a standard in this research field, however they cannot provide a direct measure of the inherently subjective feelings that participants experience. At best they provide a correlate and participants may have been using some other intrinsic metric to provide responses rather than the one which the questionnaires are appearing to target [47]. For instance, based on the present results it can be debated whether or not ownership of the red cursor ball occurred beyond just holding an object in the hand. Findings from the Rubber Hand Illusion paradigm would argue against it, since it has been reported that the more the fake hand or body looks like a human hand or body the stronger the effect of ownership as a result of visuotactile stimulation is likely to be (e.g. [14, 48, 49]). This would argue against the embodiment of the red cursor ball, which does not look like a hand at all. However, other studies have found ownership of an invisible hand or body [16, 17], a table top [50] and a hand-held tool [39], thus suggesting the existence of a perhaps more fuzzy form of ownership for which a human appearance is not necessary and which is much more flexible and could include the embodiment of tools. To our knowledge, the idea of a human-like appearance being a determining factor to ownership comes particularly from studies using the Rubber Hand or Full Body Illusion paradigms in which such comparisons were made (e.g. [14, 48, 49]). In these studies participants were generally instructed not to move their real hand or body and the illusion-inducing multisensory correspondence was created by external stimulation such as brush strokes to both the real and the dummy hand. However, keeping the body or hand perfectly still is perhaps not a very natural state to be in for a prolonged period of time and self-generated sensations through active body movements are likely more natural ways to experience multisensory correspondences involving our limbs. So the question arises to what extent the sense of ownership may be more forgiving with respect to visual appearance of the hand or tool in free movement conditions. In relation to this idea and beyond the study of ownership per se, it has for instance been shown that short exposures of tool-use or adapting to visuomotor discrepancies, in which feedback about the location of the hand is provided as an abstract dot, can lead to a morphological update of the body schema as measured using a two-point discrimination task on the skin of the arm [51–53]. This indicates that at least the metrics of our body representation are relatively flexible and do not require a humanlike appearance of the visual feedback. These findings from body schema and sensorimotor control studies would be relatively hard to reconcile with the extreme view that a human-like appearance is necessary for any form of embodiment to occur and are more consistent with the fuzzy more flexible form of ownership that can include the embodiment of tools and possibly abstract shapes. In short, looking across several directions of research it does not necessarily seem impossible for the red cursor ball in the present study to become embodied to some extent, which would correspond to the subjective ratings for ownership that the participants provided. However, whether the ratings truly reflect “ownership” of the cursor ball is hard to say beyond doubt. Participants could for instance have been giving responses based on their best guess of what “ownership” could mean in the same way as participants in a study by Slater [47] based the meaning of the arbitrary idea of a “colourful day” on how pleasant they experienced that day to be. In other words, given that participants were consistent in their ratings for ownership across the two parts of the experiments, it is likely that they were using the same intrinsic metrics across the experiment in a consistent manner, but whether these intrinsic metrics should be called “ownership” is arguable. Future research could aim to include objective behavioural or physiological correlates such as the threat response (see e.g. [22, 50]) to verify the present findings, though care should be taken that these kind of measures do not interfere with the adaptation task.

A different point to consider for both questionnaire responses as well as ratings in a scaling task, is that possibly participants compare any current condition to conditions that they have recently experienced as a reference. This means that such questionnaire and scaling task results will often be relative responses that can be used to compare conditions rather than measure the subjective feelings on an absolute scale. In other words we should be careful not to over-interpret the current results as absolute levels of ownership, agency and presence that was felt in each separate condition or directly compare the results observed here to other findings in the field. For instance, despite similar ratings we cannot safely determine whether the amount of “ownership” or “embodiment” felt for the red cursor ball observed here was the same, larger or smaller than the body-ownership felt for a rubber hand in the Rubber Hand Illusion in which the hand was kept static, as participants did not experience both conditions in one controlled experiment. It would however be an interesting direction for future research to investigate how cues such as the appearance of the ‘avatar’ (hand or abstract shape) and the level at which free movement is allowed interact for the feelings of ownership and agency.

Nevertheless, the relative nature of the questionnaires and scaling tasks is appropriate to compare conditions that are conducted within the same experimental paradigm and they at least do provide insights into the qualitative subjective experience of the virtual environment. We found that within a given context (here the comparison between the No-Delay and Delay conditions) participants were quite consistent in their responses. For instance, the questionnaires and in the scaling task provided very similar ratings for each of the subjective measures, despite the subjective responses being provided in slightly different ways. Furthermore, when the delay was first introduced in the Delay Exposure Experiment participants gave very similar ratings in the scaling task as those they gave for the Delay Condition in the Methods Test Experiment (see Fig 5). Given this consistency in responses when conditions are repeated and the observed difference in responses for No-Delay and Delay conditions, we can conclude that a feedback delay leads to a reduction in at least the subjective ratings for ownership and agency which recover with prolonged exposure. This suggests that also the qualitative subjective experience of the VR environment recovers as a result of delay adaptation.

With regard to the recovery of the subjective ratings with prolonged exposure to visual feedback delays, it is of interest to note the striking resemblance between the improvements of spatial and temporal behaviour on the target-tracking task and the recovery of the subjective ratings for ownership, agency and presence. All measures seem to follow a similar trend over time (compare Figs 4 and 5). This suggest that there may be a causal link between the behavioural and subjective measures. The question is whether this is a direct causal relationship or not. Here we will discuss two possibilities. First, genuine delay adaptation leads to a shift in perceived simultaneity between the actual movements and the visual feedback of those movements [24, 33, 54–59]. As a result, with adaptation the perceptual consequences of the delay are reduced over time, thus naturally leading to both the improvement in behavioural performance and the recovery of the subjective ratings for ownership, agency and presence. In other words, both the behavioural and subjective improvements can be effects of a change in perceived simultaneity and thus the link between the two could be indirect. To promote genuine delay adaptation (i.e. a shift in perceived simultaneity) we here used the predictable target tracking task for which such genuine delay adaptation has been previously observed [33]. It is also worth noting that some participants in the present study reported that upon removal of the delay after adaptation the cursor ball seemed to move before they did. This reversal in perceived temporal order indicates that also in the present study these participants experienced a genuine shift of perceived simultaneity. If the subjective ratings for ownership, agency and presence rely on the perceived rather than the actual temporal correspondence between the senses, delay adaptation very naturally leads to the recovery of these responses.

The second possible explanation for the recovery of the subjective ratings for ownership, agency and presence with prolonged exposure to the delay is that these subjective ratings are more directly linked to the improvement in task performance. It is possible that the ratings for ownership, agency and presence improve as a result of the behavioural improvement in the task, regardless of how the behavioural improvement came about. It has for instance been shown that even in the absence of a change in perceived simultaneity between the actual movements and the visual feedback of the movements, participants can learn to partially cope with a feedback delay by adopting a different behavioural strategy in the tracking task [33, 34]. In this case, however, the learned strategy can be readily abandoned when no longer needed and no adaptation aftereffects tend to be observed. Therefore to gain some insights into whether a behavioural task improvement by learning a strategy can explain the present results, it is worthwhile to look at the aftereffects that occur when the delay is removed. If dominated by a strategy there should be no reason why participants could not simply switch back to the strategy they used before the delay was introduced, both for the behavioural task as well as for their responses in the scaling task. However, we found that behaviourally there was a significant aftereffect for the spatial errors in the target tracking task and a similar trend (though not significant after correcting for multiple comparisons) was visible for our subjective measures. The smaller effects upon delay removal are likely due to de-adaptation effects generally being smaller and therefore it is harder for further derivative measures to reach significance. In any case, based on the observed aftereffects and trends upon removal of the delay as well as the anecdotal evidence for a reversal in perceived temporal order described above, it seems unlikely that participants were simply only using a strategy to improve in the task.

In short, the current results tentatively favour the interpretation that both the behavioural improvement in the task as well as the improvement in the subjective ratings for ownership, agency and presence may be due to a shift in the perceived simultaneity between the actual and seen movements as a result of adapting to the delay. Future work could focus on the comparison between task improvement through a change in strategy and task improvement through a genuine shift in perceived simultaneity to gain more insights into the potential causal relationship between behavioural task performance and the subjective ratings for ownership, agency and presence in VR.

## Conclusion

The results of the present study demonstrate that with prolonged exposure to feedback delay in VR both behavioural task performance and the subjective ratings for ownership and agency improve over time. In other words, though previous work showed that feedback delays attenuate the subjective ratings for ownership and agency, the present results show that these ratings can be recovered simply by exposing the participants to the delay over a longer period of time. Taken together with previous findings in the literature, this suggests that subjective ratings for ownership and agency may depend more on the perceived simultaneity between the senses, which can change as a result of delay adaptation, rather than the actual temporal discrepancy. These results will have large consequences for how VR can be used as a tool in situations in which behavioural data needs to be transmitted over a network which generally increases the feedback delay. Moreover, these results show that subjective ratings for ownership and agency are not fixed given the sensory information, but are themselves subject to adaptive changes.

## Supporting information

S1 Table Questions used to measure ownership, agency and presence. Top: Adjusted questionnaire for ownership and agency (adapted from [12]). Each item was assessed on a 7-point Likert scale from -3 (strongly disagree) to 3 (strongly agree). The questions were presented in intermixed fashion. Presence was measured using the IPQ questionnaire [29] without modification and therefore is not reproduced here. These questionnaires were used after the No-Delay and Delay conditions in the Methods Test Experiment. Bottom: Questions used in the scaling task performed after each trial in both the Method Test Experiment and the Delay Exposure Experiment. For each question participants placed a marker on a continuous scale between -1 (“not at all”) and +1 (“very much”).
(PDF)
