# Supplementary material for: Effects of prolonged exposure to feedback delay on the qualitative subjective experience of virtual reality
Source: PLoS One. 2018 Oct 24;13(10):e0205145. doi: 10.1371/journal.pone.0205145 (PMC6200207; doi:10.1371/journal.pone.0205145)
Supplement: S1 Table — Top: Adjusted questionnaire for ownership and agency (adapted from [12]). Each item was assessed on a 7-point Likert scale from -3 (strongly disagree) to 3 (strongly agree). The questions were presented in intermixed fashion. Presence was measured using the IPQ questionnaire [29] without modification and therefore is not reproduced here. These questionnaires were used after the No-Delay and Delay conditions in the Methods Test Experiment. Bottom: Questions used in the scaling task performed after each trial in both the Method Test Experiment and the Delay Exposure Experiment. For each question participants placed a marker on a continuous scale between -1 (“not at all”) and +1 (“very much”). (PDF) [file pone.0205145.s001.pdf]

Adjusted questionnaire for ownership and agency in the Methods Test Experiment

Category	Questions
Ownership	. . . I felt as if I was looking at a tool in my own hand
	. . . I felt as if the red ball was part of my body
	. . . It seemed as if I were sensing the movement of my hand in the location where the red ball moved
	. . . I felt as if the red ball was attached to my hand
Ownership Control	. . . I felt as if my real hand were turning rubbery
	. . . It seems as if I had more than one right hand
	. . . It appeared as if the red ball were drifting towards my real hand
	. . . It felt as if I had no longer a right hand, as if my right hand had disappeared
Agency	. . . The red ball moved just like I wanted it to, as if it was obeying my will
	. . . I felt as if I was controlling the movements of the red ball
	. . . I felt as if I was causing the movement I saw
	. . . Whenever I moved my hand I expected the red ball to move in the same way
Agency Control	. . . I felt as if the red ball was controlling my will
	. . . I felt as if the red ball was controlling my movements
	. . . I could sense the movement from somewhere between my real hand and the red ball
	. . . It seemed as if the red ball had a will of its own

Questions used in the scaling task in both experiments

Category	Questions
Ownership	. . . how much did you feel like the 'avatar' was part of your body?
Agency	. . . how much did it feel like you were in control of the 'avatar'?
Presence	. . . How present did you feel in the virtual environment?
